# Therapeutic Notes

**Published:** 1900-10

**Authors:** Nelson W. Wilson

**Affiliations:** Buffalo, N. Y.


### THERAPEUTIC NOTES.

Conducted by NELSON W. WILSON, M. D., Buffalo, N. Y.

### PNEUMONIA.

DR. J. A. GRACEY, in the *Charlotte Med. Jour.*, reports creosote carbonate (creosotal) of great value in pneumonia in all stages, as well as in acute bronchitis. He got fairly good results from drop doses of beechwood creosote every two hours, but prefers creosotal because it can be given in fuller doses, is more palatable and agrees better with the stomach. Of creosotal he gave 10 to 15 minims in a simple menstruum, every two or three hours.

Dr. Charles F. Stokes, U. S. Navy, reported to the Medical Society of Kings County, several cases of pneumonia covering all stages in which creosote was administered in twelve drop doses in capsules, every two hours, with most favorable results.

To prevent its separation in solutions, creosotal should be dispensed in the following combination, according to Munk (*Pharm. Central-halle*):

| | | |
|---|---|---|
| ℞ | Creosotal | 1 to 3.0 |
| | Emulsionis oleosæ | 80.0 |
| | Syrup, althaeæ | āā 100.0 |

Add the creosotal to the emulsion before the addition of the water.

---

### NEW LOCAL ANESTHETIC FOR THE EAR.

AQUEOUS solution of cocaine and eucaine having proved so unsatisfactory when applied to the tympanic membrane as anesthetics, the use of aniline oil and alcohol as vehicles, as first tried by Albert A. Gray, should prove a boon to aurist and patient, as paracentesis is stated to be entirely painless five minutes after the anesthetic is employed. Ten diops of the following preparation are injected into the external auditory canal and allowed to flow to the membrane:

| | | |
|---|---|---|
| ℞ | Hydrochlorate of cocaine . . . . . . . . . | 5 parts. |
| | Dilute alcohol . . . . . . . . . . . . . . . . | 50 " |
| | Aniline oil . . . . . . . . . . . . . . . . . . | 50 " |

Granulations of the tympanum can also be removed painlessly, first cleansing and drying the parts before using the solution. In case of thickened and hardened membranes the following formula is more effective:

| | | |
|---|---|---|
| ℞ | Hydrochlorate of cocaine . . . . . . . . . | 10 parts. |
| | Absolute alcohol . . . . . . . . . . . . . . | 30 " |
| | Aniline oil . . . . . . . . . . . . . . . . . . | 70 " |

—*Medical News.*

TRIONAL MIXTURE.

THE *Jour. Am. Med. Association* credits the following to M. A. H. Thelberg, of New Yoik:

"One of the objections to trional is its difficulty of solution and also its unpleasantness of administration when given in the usual method. The following will be found an excellent method of making a palatable mixture :

| | | | |
|---|---|---|---|
| ℞ | Trional . . . . . . . . . . . | ʒij. | 8.00 |
| | Ol. amygdal. dulc. . . . . . . . . . . . | ℥j. | 32.00 |
| | Gum acac. . . . . . . . . . . . . . . | ʒiv. | 16.00 |
| | Syrup tolutan . . . . . . . . . . . . | ℥j | 32.00 |
| | Aquæ aurant. concent. . . . . . . . . | ʒiv. | 16.00 |
| | Aquæ pur., q. s. ad. . . . . . . . . . . | ℥iv. | 128.00 |

M. Ft. emulsion. In divided doses.

"Given in this way the hypnotic effect is as pronounced, and far more readily obtained than in the present method of administering the drug."

TOPICAL APPLICATION FOR BURNS.

AFTER having washed the part with boric solution and having evacuated the blisters, if there are any, apply with the aid of a brush the following solution:

| | | | |
|---|---|---|---|
| ℞ | Tannin . . . . . . . . . . . . . . . | ʒiiss. | 10.00 |
| | Alcohol . . . . . . . . . . . . . . . | ʒiiss. | 10.00 |
| | Sulphuric ether . . . . . . . . . . . | ℥iiss. | 80.00 |

M. External use.

—*Bull. gen. de Therapeutique.*

### SODIUM PHOSPHATE IN URTICARIA.

WOLFF gives the phosphate of soda internally, every three hours, in doses of sixty to seventy-five grains, or less in infants, to cure urticaria within the space of twenty-four hours. Locally he prescribes at the same time, as a lotion, a mixture often employed in America for eczema:

| | | | |
|---|---|---|---|
| ℞ | Calamine prep. } Zinc oxidi . . } . . . . . . . . . . | āā ʒiss. | 6.00 |
| | Ac. carbolici . . . . . . . . . . . | ʒss. | 2.00 |
| | Aquæ calcis . . . . . . . . . . . . | ʒxv. | 60.00 |
| | Aquæ rosæ . . . . . . . . . . . . | ℥ivss. | 144.00 |
| M. | | | |

In infants the quantity of carbolic acid ought to be reduced according to age. In chronic urticaria the phosphate of soda taken after each meal in the dose mentioned causes uniformly a rapid relief, but relapses often occur; indeed, the patients ought to continue the use of this drug until the tendency to relapse has entirely disappeared. Sodium phosphate is particularly efficacious in urticaria of a gastro-intestinal origin, which is often the case.—*Medical News.*

---

### TOBACCO HEART.

FOR that very prevalent condition known as tobacco heart, Stern recommends the following combination:

| | | | |
|---|---|---|---|
| ℞ | Adonidin . . . . . . . . . . . . . . | gr. $\frac{1}{12}$. | .005 |
| | Ammonii carb. . . . . . . . . . . . | gr. ii. | .125 |
| | Camphoræ . . . . . . . . . . . . . . | gr. ss. | .03 |
| S.—t. i. d. | | | |

—*Merck's Archives.*

---

### SIMPLE CONJUNCTIVITIS.

In acute cases Ohlemann advises cold compresses and one of these lotions:

| | | | |
|---|---|---|---|
| ℞ | Zinci sulphat. . . . . . . . . . . . . | gr. $\frac{2}{5}$ | .026 |
| | Aquæ dest. . . . . . . . . . . . . . . | ʒiv. | 16.00 |

| | | | |
|---|---|---|---|
| ℞ | Plumbi acetat. neutralis . . . . . . . | gr. $\frac{1}{5}$ | .013 |
| | Aquæ dest. . . . . . . . . . . . . . . | ʒiiss. | 10.00 |

In chronic cases in which the epithelium of the cornea is intact, 6 to 10 drops of a 15 per cent. solution of lead water in a wineglass full of boiled water should be applied on compresses to the eye two or three times a day for fifteen minutes at a time. The following ointment is very efficacious:

| | | | |
|---|---|---|---|
| ℞ | Hydrargyri oxidi flav. . . . . . . . . | gr. iii. | .195 |
| | Vaselini . . . . . . . . . . . . . . . | ʒiiss. | 10.00 |

When additional redness of the edge of the lids is present, washes are sometimes not well borne. In such cases the following ointment should be used at night:

| | | | |
|---|---|---|---|
| ℞ | Hydrargyri precip. alb. . . | gr. iii.—iv. | .195—.260 |
| | Zinci oxidi alb. . . . . . . | gr. iv.—viiss. | .260—.500 |
| | Aceti plumbi . . . . . . | gtt. iv.—vi. | .260—.390 |
| | Vaselini . . . . . . . . | ʒvi. | 24.00 |

In chronic cases of an eczematous nature this ointment is useful:

| | | | |
|---|---|---|---|
| ℞ | Icthyol . . . . . . . | gr. $\frac{3}{10}$—$\frac{7}{10}$ | .019—.045 |
| | Amylis. | | |
| | Zinci oxidi . . . . āā | ʒiiss. | 10.00 |
| | Vaselini . . . . . . . | ʒvi.—gr. xxv. | 25.66 |

### IRRITATING COUGH OF PHTHISIS.

WHEN not accompanied by much expectoration, the following mixture is recommended:

| | | | |
|---|---|---|---|
| ℞ | Codeinæ . . . . . . . . . . . . . . | gr. iv. | .26 |
| | Acid hydrochlorici dil. . . . . . . . | ʒss. | 2.00 |
| | Sp. chloroformi . . . . . . . . . . | ʒiss. | 6.00 |
| | Syr. limonis . . . . . . . . . . . . | ℥i. | 32.00 |
| | Aquæ dest. q. s. ad. . . . . . . . . | ℥iv. | 128.00 |

M.—Ft. emulsio. Sig.—One teaspoonful at short intervals when cough is troublesome.—MURRELL.

### ACUTE CYSTITIS.

GOODELL, in the *Dominion Medical Monthly*, speaks very highly of the following prescription in the treatment of acute cystitis:

| | | | |
|---|---|---|---|
| ℞ | Atropiæ sulf. . . . . . . . . . . . | gr. i. | .065 |
| | Acid acetici . . . . . . . . . . . | gtt. xx. | 1.33 |
| | Alcoholis. | | |
| | Aquæ . . . . . . . . . . . . . . āā | ℥ss. | 16.00 |

M. Sig.—Four drops in water before each meal.

### CROUP.

JOSEPH HOLT, of New Orleans, suggested the following treatment of croup, and it has been personally prescribed many times with the happiest results:

| | | | |
|---|---|---|---|
| ℞ | Chloralis . . . . . . . . . . . . | gr. lxxv. | 5.00 |
| | Potassii bromid . . . . . . . . . . | gr. xlv. | 3.00 |
| | Amonii bromid . . . . . . . . . . | gr. xxx. | 2.00 |
| | Aqua cinnamon . . . . . . . . . . | ℥ii. | 64.00 |

M. Sig.—Teaspoonful and repeat in twenty minutes if not relieved.

"This is intended for a child about 5 years old. For younger children the dose is diminished. The chloral relieves the spasm of the larynx and the bromids allay the nervousness; so that the little patient is soon asleep, and wakes the next morning as well as usual. The first dose generally gives relief."

---

### CONSTIPATION IN CHILDREN.

A MOST effective remedy is the following:

| | | | |
|---|---|---|---|
| ℞ | Pulv. rhei . . . . . . . . . . . | gr. xxiiss. | 1.50 |
| | Pulv. ipecac . . . . . . . . . . | gr. iv. | .25 |
| | Sodii bicarb. . . . . . . . . . | gr. lx. | 4.00 |
| | Syr. simp. . . . . . . . . . . | ℥iv. | 128.00 |

M. Sig.—Teaspoonful every three hours.

---

### ACUTE GASTRITIS.

DR. PEPPER'S treatment for the vomiting of acute gastritis was one of the following prescriptions:

| | | | |
|---|---|---|---|
| ℞ | Hydrarg. chlor. mite . . . . . . . | gr. i. | .065 |
| | Bismuth subnitrat. . . . . . . . . | gr. xxxvi. | 2.39 |

M.—Ft. powders xii. Sig.—Take one powder dry upon the tongue every three hours until four or five have been taken.

| | | | |
|---|---|---|---|
| ℞ | Acid carbolic . . . . . . . . . . . | gtt. iv. | .26 |
| | Sodii bicarb. . . . . . . . . . . . | ʒiss. | 6.00 |
| | Elix. aurant. . . . . . . . . . . . | ℥ss. | 16.00 |
| | Aquæ q. s. ad. . . . . . . . . . | ℥iv. | 128.00 |

M. Sig.—A teaspoonful every three hours.

---

### GONORRHEA, INTRAURETHRAL INJECTION.

POUSSON reports favorable results from the use of the following, the formula being based on the principle that a mixture of several antiseptics exhibits more energetic therapeutic action than any single one of the same even in strong solution:

| | | | |
|---|---|---|---|
| ℞ | Menthol . . . . . . . . . . . . . | gr. ⅓ | .022 |
| | Acidi salicylici . . . . . . . . . . | gr. iss. | .100 |
| | Acidi carbolici . . . . } | | |
| | Acidi lactici . . . . . } āā . . . . | gr. iii. | .200 |
| | Ol. eucalypti . . . . . } | | |
| | Methyl salicylatis . . } | | |
| | Resorcin . . . . . . . . . . . . . | gr. viii. | .520 |
| | Aq. dest. . . . . . . . . . . . . | ℥iii. | 96.00 |

M. Sig.—External use.

DYSMENORRHEA.

| | | | |
|---|---|---|---|
| ℞ | Ergotin . . . . . . . . . . . . . . . | gr. xv. | 1.00 |
| | Quinine sulphat. . . . . . . . . . . . | gr. iii. | .20 |
| | Pulv. digitalis fal. . . . . . . . . . . | gr. iss. | .10 |
| | Pulv. coca, q. s. | | |

M.—For ten pills. Sig.—Three to five may be taken daily.

—*Presse Medicale.*

